# Objectively assessed recess physical activity in girls and boys from high and low socioeconomic backgrounds

**DOI:** 10.1186/1471-2458-14-192

**Published:** 2014-02-21

**Authors:** Georges Baquet, Nicola D Ridgers, Aurélie Blaes, Julien Aucouturier, Emmanuel Van Praagh, Serge Berthoin

**Affiliations:** 1 University of Lille Nord de France, UDSL, EA 4488 "Physical Activity, Muscle Health", Lille, France; 2 Centre for Physical Activity and Nutrition Research, Deakin University, Melbourne, Australia; 3 Blaise Pascal University, EA 3533, Clermont-Ferrand, France

**Keywords:** Children, Accelerometry, Socio-economic status, Behavior

## Abstract

**Background:**

The school environment influences children’s opportunities for physical activity participation. The aim of the present study was to assess objectively measured school recess physical activity in children from high and low socioeconomic backgrounds.

**Methods:**

Four hundred and seven children (6–11 years old) from 4 primary schools located in high socioeconomic status (high-SES) and low socioeconomic status (low-SES) areas participated in the study. Children’s physical activity was measured using accelerometry during morning and afternoon recess during a 4-day school week. The percentage of time spent in light, moderate, vigorous, very high and in moderate- to very high-intensity physical activity were calculated using age-dependent cut-points. Sedentary time was defined as 100 counts per minute.

**Results:**

Boys were significantly (p < 0.001) more active than girls. No difference in sedentary time between socioeconomic backgrounds was observed. The low-SES group spent significantly more time in light (p < 0.001) and very high (p < 0.05) intensity physical activity compared to the high-SES group. High-SES boys and girls spent significantly more time in moderate (p < 0.001 and p < 0.05, respectively) and vigorous (p < 0.001) physical activity than low-SES boys.

**Conclusions:**

Differences were observed in recess physical activity levels according to socioeconomic background and sex. These results indicate that recess interventions should target children in low-SES schools.

## Background

The school environment influences children’s opportunities for physical activity (PA) [1]. It is suggested that schools in high socioeconomic areas have higher quality and better PA facilities than schools in less affluent areas. Inchley et al. [2] have found an association between lower socioeconomic status (SES) and lower levels of primary aged children’s physical activity, whilst adolescents from lower SES groups consistently reported lower levels of vigorous physical activity than those from higher SES groups, notably for the girls. Compared with boys of higher SES, boys of lower SES were more active because they devoted more time to moderate intensity activities [3]. To the contrary, Spinks et al. [4] showed no association between children’s daily physical activity and SES and Aaron et al. [5] concluded that SES was not found to be a determinant of activity levels in adolescents. As Cauley et al. [6] reported that SES could impinge differently on separate manifestations of activity; future research needs to identify how SES affects physical activity levels.

Whilst differences have been reported in daily physical activity levels between low and high SES groups, a recent review has reported inconclusive effects of SES on children’s recess physical activity [7]. Parrish et al. [8] investigated the possible associations of SES with PA using observational data, finding no significant differences between the effects of different SES on children’s playground activity levels. However, as PA opportunities do not seem to be equal across socioeconomic groups, it is possible that more emphasis should be placed on increasing PA among children from low socioeconomic group, especially in girls who are typically less active in this context [7]. It could be argued that sociocultural factors may influence recess activity levels [9] and sex differences could be due to the fact that school environments are better adapted for boys than girls, rather than biological or social variations [10]. Due to the potential contribution of recess to daily PA recommendations, it is important to maximize the opportunities when children are active for those who may have limited opportunities for PA based on SES background.

Consequently, given the paucity of research that has examined SES effects on boys’ and girls’ physical activity within specific times of the day, such as recess, further research is needed to establish whether differences occur. Outside of school children from low SES areas have been found to be less active [11]. Therefore recess may provide an opportunity to increase their PA levels as a safe environment conducive for PA can be provided (markings, equipment etc.). Prior to intervening, it seems important to know how active they are during this time compared to high SES peers. Such information would further inform intervention efforts in the future and enable researchers to systematically investigate the effects of PA school-based interventions separately in lower and higher SES groups in the future [12].

The primary purpose of this study was to determine objectively school recess PA in 6-to-11-yr-old girls and boys from high and low socioeconomic background. It was hypothesized that school recess PA would be higher in high-SES area than in low-SES area.

## Methods

### Participants

Four elementary schools located in the same geographical area in the north of France were recruited to participate in the study. They had similar playground space (around ~ 1300 m^2^ and 1500 m^2^). Two schools were located in low-SES areas and the remaining 2 schools were in high-SES areas. This classification was obtained from the Ministry of National Education following children’s school performances and socio-economical parameters. It was based on the economic and academic profile of the catchment area of the schools the children attended (two gross-income groups corresponding to low- and high-SES areas). The performances and socio-economical parameters are the underachievement in schools, the number of scholarships, the socio-economic structure of the area, the socio-professional category, the unemployment rate, the number of families, the proportion of foreigners, the housing density, the frequency of placement of children, the number of educational assistance interventions and the number of equipment and services (leisure centers, youth centers). Four hundred and seven children (201 girls and 206 boys) aged 6 to 11 years old and their guardians gave informed written consent to participate. The low-SES group (low-SES) included 222 children (102 girls and 120 boys) and the high-SES group (high-SES) consisted of 185 children (99 girls and 86 boys). The study was designed in accordance with ethical standards of the Helsinki Declaration of 2008 and received approval from the “Comité Consultatif de Protection des Personnes en Recherche Biomédicale de Lille”.

### Anthropometric measurements

Height was measured to the nearest 0.1 cm with a wall stadiometer (Vivioz medical, Paris, France) and body mass was measured to the nearest 0.1 kg with a calibrated electronic balance (Tanita TBF 543, Tanita Inco, Iokyo, Japan). Body Mass Index (BMI) was calculated according to equation: BMI = body mass (kg)/height ^2^ (m).

### Physical activity monitoring

Children’s PA was assessed with a uniaxial accelerometer (The ActiGraph®, Manufacturing Technologies, Inc., model GT1M), during school recess time (morning and afternoon) over 4 school days (Monday, Tuesday, Thursday, Friday). Children wore the accelerometer on the right hip fastened with an elastic belt. In France, primary school children experience, on average, up to 288 recess periods per year, 36 weeks per year, each recess lasting about 15 min. The ActiGraph device facilitates the measurement of human movement (frequency and intensity) over a user-specified time epoch. In this study, the epoch was set at 2-s [13]. Accelerometers were distributed in the morning when the children arrived at school and were returned after the afternoon recess period. Data were then downloaded for statistical analysis at the end of each day.

### Data reduction

To be included in the analyses, children needed to have worn the monitors during each recess (morning and afternoon) on the 4 days of data collection. Age-specific count cut-points corresponding to Light (LPA; ≤3.99 METs); Moderate (MPA; 4.00 METs - 5.99 METs); Vigorous (VPA; ≥6.00 METs); and Very high (VHPA; ≥9.00 METs) were derived from the METs prediction equation, i.e., METs = 2.757 + (0.0015 × counts.min^-1^) – (0.08957 × age [year]) – (0.000038 × counts.min^-1^ × age [year]) (r = 0.74) [14]. MPA, VPA and VHPA were summed to obtain time spent in moderate- to vigorous-intensity physical activity (MVPA). A threshold of 4 METs was chosen to represent MPA as brisk walking has been associated with an energy cost of 4 METs in calibration studies [13]. A cut-point of 100 counts.min^-1^ was used for sedentary activity as it exhibited excellent classification accuracy [14] and is a good estimate of free-living sitting time [15]. To compare the time spent in different PA levels between groups, PA time is reported as the percentage of total recess time.

### Statistical analysis

Forty-six children (22 girls and 24 boys) had incomplete data (2 recess periods a day for the 4 days of monitoring) and were removed from the dataset. A sample of 361 children (179 girls and 182 boys) was retained for the statistical analyses. The low-SES group included 197 children (90 girls and 107 boys) and the high-SES group 164 children (89 girls and 75 boys). All values are expressed as mean ± standard deviation (mean ± SD). Data were analyzed using a two-way ANOVA (sex*SES), with the proportion of time spent in sedentary time and each of the physical activity variables as the outcome variables. Newman-Keuls post hoc tests were carried out when the ANOVA analyses revealed a significant difference or interaction between sex and SES. Significance was set at p < 0.05. Statistical analyses were undertaken using Statistica 6 software (StatSoft Inc, Paris, France).

## Results

Descriptive age and anthropometric data of the children are presented in Table 1. Body Mass Index in children from low-SES was significantly (p < 0.05) higher when compared to high-SES. No other differences in anthropometric data were observed.

**Table 1:** Mean ± SD anthropometric measurements of children

	**Age (years)**	**Body mass (kg)**	**Stature (cm)**	**Body mass index (kg.m**^**-2**^**)**
Whole group (n = 361)	8.8 ± 1.5	31.2 ± 8.1	132.8 ± 9.6	17.4 ± 2.7
Boys (n = 182)	8.8 ± 1.9	31.5 ± 8.3	133.5 ± 9.2	17.4 ± 2.8
Girls (n = 179)	8.8 ± 1.5	30.9 ± 7.9	132.2 ± 9.9	17.4 ± 2.6
High-SES group (n = 160)	8.7 ± 1.6	30.3 ± 7.5	133.3 ± 9.5	17.1 ± 2.3
Low-SES group (n = 172)	8.9 ± 1.4	31.9 ± 8.5	132.3 ± 9.6	17.7 ± 2.9*

high-SES: high socio-economic status; low-SES: low socio-economic status.

^*^: significantly different between high-SES and low-SES groups at p < 0.05.

Significant main effects were observed by SES group for the proportion of time spent in LPA, MPA, VPA and MVPA (Table 2). Low-SES children engaged in significantly more LPA (+4.5%; p < 0.001) and VHPA (+1.7%; p < 0.01) but significantly less MPA (-2.6%), VPA (-3%) and MVPA (-4%) (all p < 0.001) than high-SES children. A sex main effect on PA was also observed (Table 2). Boys spent significantly (p < 0.001) more time than girls in MPA (+1.8%), VPA (+1.4%), VHPA (+5.1%) and MVPA (+8.4%), while girls spent significantly (p < 0.001) more time in sedentary (+7.5%).

**Table 2:** Recess time spent at different physical activity levels according to sex and socioeconomic status (%, mean ± SD)

	**Sex**		**SES**		**Interaction**
	**Boys (n = 182)**	**Girls (n = 179)**	**low-SES (n = 197)**	**high-SES (n = 164)**	Sex*SES
Sedentary	38.6 ± 12.3	46.1 ± 10.7***	41.8 ± 11.1	42.9 ± 13.3	ns
LPA	29.6 ± 6.2	30.7 ± 6.1	32.1 ± 5.6°°°	27.6 ± 5.9	ns
MPA	12.3 ± 4.9***	10.5 ± 4.8	10.2 ± 3.6	12.8 ± 5.8°°°	p < 0.05
VPA	5.6 ± 4.1***	4.2 ± 3.2	3.5 ± 2.2	6.5 ± 4.5°°°	p < 0.001
VHPA	14.1 ± 6.7***	9.0 ± 5.4	12.4 ± 5.4°°	10.7 ± 7.7	ns
VPA + VHPA	19.7 ± 8.2***	13.2 ± 6.2	15.9 ± 6.3	17.2 ± 9.5	p < 0.05
MVPA	32.1 ± 11.1***	23.7 ± 9.1	26.1 ± 8.6	30.1 ± 12.9°°°	p < 0.01

SES: socio economic status; high-SES: high socio-economic status; low-SES: low socio-economic status; LPA: light physical activity; MPA: moderate physical activity; VPA: vigorous physical activity; VHPA: very high physical activity; MVPA: moderate to very high physical activity.

^***^: significantly different between boys and girls at p < 0.001. °°: significantly different between high-SES and low-SES at p < 0.01; °°°: at p < 0.001.

The statistical analyses showed a significant sex*SES interaction for MPA, VPA and MVPA (Table 2). No difference was found between low-SES and high-SES for sedentary time. Children from the low-SES group spent significantly more time than children from the high-SES group in LPA (+4.5%, p < 0.001) and VHPA (+1.7%, p < 0.05). Boys from high-SES spent significantly more time in MPA, VPA and MVPA (+4.0%, +4.3% and +7.7%, respectively; all p < 0.001) than boys from low-SES. Girls from high-SES spent significantly more time in MPA (+1.7%, p < 0.05) and VPA (+2.0%, p < 0.001) than girls from low-SES (Table 3).

**Table 3:** Recess time spent at different physical activity levels by sex and socioeconomic status (%, mean ± SD)

	**Boys**		**Girls**	
	**Low-SES (n = 107)**	**High-SES (n = 75)**	**Low-SES (n = 90)**	**High-SES (n = 89)**
MPA	10.7 ± 3.2	14.7 ± 5.9***	9.6 ± 4.1	11.3 ± 5.3*
VPA	3.8 ± 1.7	8.1 ± 5.2***	3.2 ± 2.7	5.2 ± 3.4***
VPA + VHPA	18.2 ± 5.9	22.0 ± 10.3**	13.2 ± 5.6	13.2 ± 6.7
MVPA	28.9 ± 8.0	36.6 ± 13.3***	22.9 ± 8.4	24.5 ± 9.6

SES: socio economic status; high-SES: high socio-economic status; low-SES: low socio-economic status; LPA: light physical activity; MPA: moderate physical activity; VPA: vigorous physical activity; VHPA: very high physical activity; MVPA: moderate to very high physical activity.

^*^: significantly different between SES at p < 0.05; ^***^, at p < 0.001. °: significantly different between gender at p < 0.05; °°° at p < 0.001.

## Discussion

The aim of this study was examine differences in school recess physical activity in children attending schools in low and high SES areas. The major findings were that: 1) recess PA levels differed between high-SES children and low-SES children; and 2) high-SES boys and girls were more active than low-SES boys and girls.

In the present study, patterns of physical activity during school recess differed according to sex and SES. Interestingly, no significant difference was found between SES groups for sedentary time. However, low-SES children spent significantly more time in LPA (p < 0.001) and VHPA (p < 0.05) than high-SES children, while the latter spent significantly more time in MPA, VPA and MVPA (p < 0.001). Seabra et al. [11] reported that children’s attraction to PA varies in accordance with sex and SES and demonstrated a socioeconomic trend with regard to the perceived importance of participating in PA. Girls and lower SES children tend to be less active than boys and children in higher SES, which is consistent with the results of the present study. House [16] has underlined the effects of socioeconomic inequalities on children’s PA participation. Higher social classes convey positive attitudes towards PA, which influence children’s attitudes and health-related behavior. It is possible that such differences between SES backgrounds may contribute to the differences observed in this study. It is important to note that in the school context, recess provides an opportunity for children to be active and accounts for approximately one quarter of primary school day [17]. As such, recess presents an ideal opportunity to engage children’s physical activity behaviors and contributes to physical activity recommendations, and maybe an important time to increase the physical activity levels of low-SES children.

According to the review by Ridgers et al. [7], SES was not consistently related with MVPA during recess, which is not supported by the current study. However, the literature focusing on this topic is very sparse [8,18,19]. Parrish et al. [8] have investigated PA between schools from lower and average SES areas. Whilst their results indicated that 2 of the 3 most active and 4 of the 5 least active schools were rated lower SES, no significant association was found between playground MVPA and SES. Higher rates of participation in MVPA were reported in children in private school than those attending public schools [18]. These rates could be partly explained by the better finances of private schools, due to higher fees contributing to offer more opportunities to be involved in different PA during lunchtime. However, while most studies have focused on MVPA, the present study looked at all physical activity intensities and sedentary time. Given that differences between low- and high-SES backgrounds occurred at different intensities, it appears that interventions that aim to increase low-SES children’s physical activity should implement strategies that target the differences intensities.

Ridgers et al. [7] reported that access to different facilities (spaces, gyms) or providing equipment is benefit to children’s PA during recess. In the present study, all participating schools had similar playground space and the equipment did not differ between schools. This suggests that other factors of school environment could explain PA level differences, such as social exclusion or playground issues, for example. However, as no data concerning children’s actual play behavior during recess time was collected in the present study, no clear conclusions can be drawn. In addition, the contribution of recess to the children’s weekly PA could not be evaluated as children did not wear the devices outside school. It would be interesting to compare the impact of SES at school and outside school and to quantify the contribution of recess to daily physical activity. Moreover, lunchtime PA was not included in the study as children in the participating schools could eat lunch at school or go home and return throughout lunchtime. This made it difficult to determine how PA was undertaken at school.

The literature shows that living in low SES neighborhoods is related to fewer opportunities to be physically active [20,21]. Drenowatz et al. [22] reported that low-SES children had lower PA levels and spent more time in sedentary behavior than high-SES children. Kolle et al. [1] conducted two cross-sectional studies over a 5-yr period. In the first study period, the authors showed that children from low-SES groups participated in more MVPA than children from middle- and high-SES groups (p < 0.001 and p = 0.007, respectively). In the second study, there was no association between time spent in MVPA and SES. However, PA measurements in the Drenowatz et al. and Kolle et al. studies [1,22] were made both during and outside school hours. Interactions between PA and SES showed that some characteristics of the school and out-of-school environments might also influence children’s PA. Hohepa et al. [23] reported that a large proportion of teenagers were not active in low-SES area and lower participation in PA was observed during school time (recess and lunchtime) than after school. These studies suggest that it is important that low SES children have easy access to high-quality PA facilities and support at school. Debourdeaudhuij et al. [12] have stated that a PA stimulating environment was an important factor for low SES children PA compared to their counterparts in high SES areas. However, interventions to promote PA had similar effects in adolescents regardless of SES background and were not able to show a significant widening or narrowing of inequalities [12].

Gender is the most significant factor contributing to differences in PA participation during school recess [7]. Girls engage in lower levels of MVPA than boys and this gender difference increases with age. In the present study, data provided from morning and afternoon recess, excluding lunch recess, were similar than those previously reported to the literature [24,25]. Notably, boys were more active than girls irrespective of socioeconomic background and significantly more girls were sedentary. However, no gender differences were reported for LPA. The reasons for these gender differences may be attributable to the social context of recess, the structure of recess, the definition of recess and the behaviors that boys and girls engage in during this time [10]. Blatchford et al. [26] emphasized the influence of sex roles, with boys viewing recess time as an opportunity to engage in competitive games whereas girls viewing it as an opportunity to socialize with friends and then engaging in more sedentary.

While high SES boys were significantly more active than those of low-SES (p < 0.001), no difference was found for the girls. These data suggest that there is an additive effect of gender and SES. Fuchs et al. [3] reported that weekly activity time among girls did not vary substantially with socioeconomic status. However, compared with boys of higher socioeconomic status, boys in the lower socioeconomic grouping were more active because they devoted more time to moderate activities. In the present study, low-SES boys spent significantly more time in VPA, but were less active than the high-SES boys. In the study of Inchley et al. [2], children from lower SES groups also reported lower levels of VPA and girls from the highest SES groups participated in less leisure-time VPA than boys from the lowest SES groups. They suggested that girls from low SES backgrounds were at particular risk of low physical activity.

Physical activity during recess may be important in achieving children’s recommended daily physical activity. Ridgers et al. [27] recommended a health-related criterion of 40% of playtime in MVPA during recess. This criterion was not reached in this study regardless socioeconomic background, even though high SES children spent significantly more time in MVPA (p < 0.001). Overall the results of the present study seem to suggest that different strategies may be needed to increase the PA levels of boys and girls from different SES background. A recent review suggested that playground markings and non-fixed equipment may increase children’s physical activity [28], though further research is needed to establish which strategies may be beneficial across different SES groups.

## Conclusion

Boys and girls from different SES backgrounds engage in different levels of PA during school recess. These results indicate that recess interventions should target children in low-SES schools and girls to increase their physical activity levels.

## Competing interests

The authors declare that they have no competing interests.

## Authors’ contributions

GB has contributed to conception and design, acquisition of data, and analysis and interpretation of data. NDR has contributed to conception and design, and interpretation of data. AB has contributed to acquisition of data. JA, EVP and SB have been involved in drafting the manuscript or revising it critically. All authors read and approved the final manuscript.

## Authors’ information

Nicola D Ridgers is supported by an Australian Research Council Discovery Early Career Researcher Award

This work is supported by the Conseil Régional Nord-Pas de Calais (ARCIR Activité Physique et Santé de l’Enfant).

## Pre-publication history

The pre-publication history for this paper can be accessed here:

http://www.biomedcentral.com/1471-2458/14/192/prepub
